# Assessing Suitability of Co@Au Core/Shell Nanoparticle Geometry for Improved Theranostics in Colon Carcinoma

**DOI:** 10.3390/nano11082048

**Published:** 2021-08-11

**Authors:** Udesh Dhawan, Ching-Li Tseng, Huey-Yuan Wang, Shin-Yun Hsu, Meng-Tsan Tsai, Ren-Jei Chung

**Affiliations:** 1 Institute of Chemistry, Academia Sinica, 128, Sec. 2, Academia Rd., Taipei 11529, Taiwan; Udesh.dhawan@glasgow.ac.uk; 2 Department of Materials and Mineral Resources Engineering, National Taipei University of Technology (Taipei Tech), 1, Sec. 3, Zhongxiao E. Rd., Taipei 10608, Taiwan; 3 Centre for the Cellular Microenvironment, University of Glasgow, Glasgow G12 8QQ, UK; 4 Graduate Institute of Biomedical Materials and Tissue Engineering, College of Biomedical Engineering, Taipei Medical University, 250, Wu-Hsing St., Taipei 11031, Taiwan; chingli@tmu.edu.tw; 5 International Ph.D. Program in Biomedical Engineering, College of Biomedical Engineering, Taipei Medical University, 250, Wu-Hsing St., Taipei 11031, Taiwan; 6 Research Center of Biomedical Device, College of Biomedical Engineering, Taipei Medical University, 250, Wu-Hsing St., Taipei 11031, Taiwan; 7 International Ph.D. Program in Cell Therapy and Regenerative Medicine, College of Medicine, Taipei Medical University, 250, Wu-Hsing St., Taipei 11031, Taiwan; 8 Department of Stomatology, MacKay Memorial Hospital, 92, Sec. 2, Zhongshan N. Rd., Taipei 10449, Taiwan; wang461110@yahoo.com.tw; 9 Department of Chemical Engineering and Biotechnology, National Taipei University of Technology (Taipei Tech), 1, Sec. 3, Zhongxiao E. Rd., Taipei 10608, Taiwan; wendy780830jim@gmail.com; 10 Department of Electrical Engineering, Chang Gung University, 259, Wenhua 1st Rd., Taoyuan City 33302, Taiwan; mttsai@mail.cgu.edu.tw; 11 Department of Neurosurgery, Chang Gung Memorial Hospital, 5, Fuxing St., Guishan Dist., Taoyuan City 33305, Taiwan

**Keywords:** core–shell, cobalt–gold, nanoparticles, hyperthermia, methotrexate, colon carcinoma

## Abstract

The interactions between cells and nanomaterials at the nanoscale play a pivotal role in controlling cellular behavior and ample evidence links cell intercommunication to nanomaterial size. However, little is known about the effect of nanomaterial geometry on cell behavior. To elucidate this and to extend the application in cancer theranostics, we have engineered core–shell cobalt–gold nanoparticles with spherical (Co@Au NPs) and elliptical morphology (Co@Au NEs). Our results show that owing to superparamagnetism, Co@Au NPs can generate hyperthermia upon magnetic field stimulation. In contrast, due to the geometric difference, Co@Au NEs can be optically excited to generate hyperthermia upon photostimulation and elevate the medium temperature to 45 °C. Both nanomaterial geometries can be employed as prospective contrast agents; however, at identical concentration, Co@Au NPs exhibited 4-fold higher cytotoxicity to L929 fibroblasts as compared to Co@Au NEs, confirming the effect of nanomaterial geometry on cell fate. Furthermore, photostimulation-generated hyperthermia prompted detachment of anti-cancer drug, Methotrexate (MTX), from Co@Au NEs-MTX complex and which triggered 90% decrease in SW620 colon carcinoma cell viability, confirming their application in cancer theranostics. The geometry-based perturbation of cell fate can have a profound impact on our understanding of interactions at nano-bio interface which can be exploited for engineering materials with optimized geometries for superior theranostic applications.

## 1. Introduction

Nanotechnological advancements for cancer treatment have shown an abrupt increase in the past decade and engineering nanoparticles with definitive size and composition is at the core of these advancements. A plethora of nanoparticles such as iron oxide, gold, silver, and silica have found applications in cancer therapy owing to properties assisting in medical imaging or drug delivery, thereby improving the disease prognosis [1,2,3]. A majority of nanomaterials are engineered from a single element which limits their application in multiple domains. To overcome these challenges, a new class of multifunctional nanomaterials, such as core–shell nanoparticles, have been engineered comprising a mixture of different elements, which allows for the application of the same nanoparticle in different domains [4,5]. Gold nanoparticles display remarkable biocompatibility and are therefore often employed to form the shell in a core–shell nanoparticle [6,7,8]. Besides, the presence of gold also allows for the ease of functionalization with a number of chemodrugs. For instance, Methotrexate can be conjugated with iron gold core–shell nanoparticles via 2-aminoethanethiol, EDC ((1-ethyl-3-(3-dimethylamino) propyl carbodiimide, hydrochloride) or (*N*-Hydroxysuccinimide, NHS) coupling or through pH-linkers [9]. The ease of functionalization simplifies the use of nanoparticles for cancer therapy. Furthermore, gold nanoparticles have also found applications as contrast agents in medical imaging [10], allowing for simultaneous cancer imaging and chemodrug release. Gold nanoparticles have also been exploited to engineer metallic core/satellite structures and for surface-enhanced Raman spectroscopy (SERS) application [11,12,13]. All these results collectively demonstrate the suitability of gold as a crucial component while synthesizing core–shell nanoparticles for cancer therapy. These scientific explorations have led to conjugation of gold with magnetic nanoparticles to engineer biocompatible nanomaterials for hyperthermia-induced cancer treatment. 

Despite tremendous success of utilizing magnetic nanoparticles for hyperthermia induced cancer therapy, persistent challenges exist. One of these is the intrinsic nanoparticle cytotoxicity which harms the healthy stroma surrounding cancer cells [14]. Similar observations have led to identification of nanomaterial parameters such as size [15], composition [16], surface charge [17] and stability [18], confirming that the rational design and synthesis of nano-particulates with definitive size and composition is of paramount importance for a given therapeutic application. However, studies highlighting the shape-dependent nanoparticle cytotoxicity are strikingly scarce. The pathogenic recognition inside the biological systems is shape-dependent. For instance, Doshi et al. showed that macrophages operate on geometry-based target recognition [19]. Other studies have shown that the aggregation and expulsion from the physiological system is a function of the nanoparticle shape along with the size. This raises an interesting question: whether the nanomaterial cytotoxicity in-vivo is also a function of nanoparticle shape [20]. Although the reported recognitions occur at a micro-scale, it is a reasonable assumption that similar cellular recognition patterns manifest at a nano-scale. This implies that the clinical prospects of employing nanoparticles for cancer therapy can be improved by controlling its shape.

Chemotherapy can be an effective route for cancer treatment [21]. However, the physiological differences between different cancer types and non-specific course of action are crucial factors limiting the therapeutic efficiency of chemodrugs. To overcome this, researchers have turned to utilizing chemodrug-conjugated nanoparticles for targeted drug delivery [22,23]. Furthermore, concomitant material properties such as superparamagnetism allows for the nanoparticle activation in the presence of an alternating magnetic field (AMF), resulting in temperature elevation of the material’s surroundings. In this given scenario, the nanoparticles serve a dual purpose: first, the nanoparticles can transport the drug to the cancerous site by means of magnetic guidance followed by its release upon AMF induction [24,25]. Secondly, the elevated temperature also sensitizes the cancer cells to the chemodrug [26]. The dual impact results in apoptosis of the majority of the cancerous cells, reducing the chances of a relapse. The elevated temperature of 40–42 °C also poses minimal risk of protein degradation. Recent studies have shown that doping of metallic atoms such as cobalt in ferrite can lead to cancer cell death via hyperthermia [27]. The cytotoxicity displayed by ferrite is still a significant concern. However, we may be able to overcome this limitation by engineering nanoparticles via an amalgamation of cobalt and gold where the gold shell provides superior biocompatibility as compared to other metals and superparamagnetic properties presented by cobalt are exploited to generate hyperthermia for causing cancer cell apoptosis.

Employing magnetic nanoparticle-induced hyperthermia requires exposure of the entire organism to AMF. The increase in temperature of the targeted area can cause side-effects (damage to healthy stroma) [28]. Although designing organ-specific AFM coils is an option, photostimulation does not require region-specific design. Depending on tumor localization, wavelength of light can be tuned to control its penetrability, making it a more straightforward approach for site-specific cancer treatment. For this reason, scientists have turned to photo-stimulation which involves irradiation of the diseased area with a specific light wavelength, resulting in nanoparticle activation and heat generation [8]. The exposure of only the diseased area also limits the side-effects of this process.

Herein, we have addressed the following two challenges: (a) elucidate the nanoparticle shape-dependent cytotoxicity and (b) engineer a new class of multifunctional nanoparticles for medical imaging and cancer therapeutics via photo-stimulation induced hyperthermia. Briefly, iron-cobalt core–shell nanoparticles with spherical and elliptical morphology were engineered and a systematic comparison of their magnetic properties was performed. The prospect of using these nanoparticles for AMF and photo-stimulation induced hyperthermia is also explored. Using an in-vitro model, we also elucidate the effect of nanoparticle morphology on cytotoxicity. To achieve multi-functionality, we investigated the application of both nanoparticles in medical imaging. Finally, Methotrexate, an anti-cancer drug was conjugated to both nanoparticle morphologies via 2-aminoethanethiol grafting. The efficacy of nanoparticles in triggering cancer cell apoptosis via AMF or photo-stimulation-induced hyperthermia is also explored.

## 2. Materials and Methods

### 2.1. Reagents

Dulbecco’s Modified Eagle Medium and fetal bovine serum (FBS) were purchased from Gibco (Thermo Fisher Scientific, Waltham, MA, USA). Cobalt Hexahydrate, hydrogen tetrachloroaurate (III) trihydrate (HAuCl_4_·3H_2_O) and ethylene chloride were purchased from Alfa Aesar (Heverhill, MA, USA). Cethyltrimethylammonium bromide (CTAB) and 1-Butanol were purchased from Sigma-Aldrich (St. Louis, MO, USA). Isooctane was purchased from Tedia (Fairfield, OH, USA). Methotrexate (MTX), sodium borohydride were purchased from Acros organics (Fair Lawn, NJ, USA), silver nitrate (AgNO_3_), ascorbic acid and citric acid were purchased from Honeywell Riedel-de Haen (Seelze, Germany). 2-Aminoethanethiol was purchased from Tokyo Chemical Industry (TCI, Tokyo, Japan). All other chemicals of analytical grade were purchased from Sigma (St. Louis, MO, USA).

### 2.2. Synthesis, Purification and Characterization of Co@Au Nanoparticles

To prepare a microemulsion of Co(NO_3_)_2_·6H_2_O and NaBH_4_, equal volumes of CTAB, 1-Butanol and isooctane were mixed together and 0.245 × 10^−3^ M Co(NO_3_)_2_·6H_2_O and 2.9 × 10^−3^ M NaBH_4_ were added to this mixture. The weight percent of aqueous solution, CTAB, 1-Butanol and isooctane were 21%, 18%, 53% and 8%, respectively. Additionally, gold microemulsion and reducing agent were prepared using equal volumes of CTAB, 1-Butanol and isooctane followed by addition of 0.2 M HAuCl_4_·3H_2_O and 0.8 M NaBH_4_. The weight percent of aqueous solution, CTAB, 1-Butanol and isooctane were 17.3%, 14.5%, 57.8% and 10.4%, respectively. To synthesize Co@Au Nanoparticles (Co@Au NPs), the Co (NO_3_)_2_·6H_2_O microemulsion was placed in a three-necked flask and purged with argon for 5 min. The Co and HAuCl_4_·3H_2_O microemulsions were sequentially injected via a syringe. The microemulsions were allowed to stand together in argon atmosphere for 30 min to obtain Co@Au magnetic NPs. The excess surfactant and Au NPs were washed with 99.8% ethanol and centrifuged thrice at 9000 rpm for 10 min. The magnetic nanoparticles were dried using a vacuum and collected using a permanent magnet. X-ray diffraction (XRD, XRD-6000, Shimadzu, Kyoto, Japan) was performed to confirm the core–shell structure of the nanoparticles while Energy dispersive X-ray spectroscopy (EDS, X-Max 80 mm, Oxford Instruments, Oxford Instruments, Abingdon, UK) and superconducting quantum interference device (SQUID, MPMS7, Quantum Design, San Diego, CA, USA) were performed to analyze the composition and magnetic properties of the nanoparticles, respectively.

### 2.3. Synthesis, Purification and Characterization of Co@Au Nanoparticles with Elliptical Morphology

The nanoparticle growth solution was prepared using 200 mM CTAB (5 mL), 7.8 mM HAuCl_4_·3H_2_O (5 mL), 40 mM AgNO3 (0.9 mL) and 80 mM ascorbic acid (0.8 mL). One milligram of Co@Au nanoparticles were then mixed with 2.5 mM aqueous citric acid (0.25 mL). The Co@Au solution was dispersed into the nanoparticle growth solution in dark for 1 h. The solution was then centrifuged at 9000 rpm for 10 min and washed thrice using 99.8% ethanol to obtain Co@Au Nanoparticles with elliptical morphology (Co@Au NEs). The optical properties were studied using UV-Vis spectrophotometer (2100 pro, GE Healthcare Life Sciences, Piscataway, NJ, USA).

### 2.4. Surface Modification of Co@Au NPs and Co@Au NEs with Anti-Cancer Drug

The surface modification of spherical (NPs) and elliptical (NEs) nanoparticles was performed in a two-step procedure. In step one, NPs/NEs and 2-aminoethanethiol were dissolved in Deionized (DI) water in a ratio of 1:10 followed by uniform dispersion using an ultrasonicator for 30 min. The mixture was then stirred for 24 h at 60 °C. The mixture was then centrifuged at 9000 rpm for 10 min and washed thrice with ethanol followed by vacuum drying to obtain functionalized NPs or NEs. In step two, 10 mg MTX was dissolved in 10 mL Phosphate-buffered saline (PBS) (pH 11). Then, 2-aminoethanethiol functionalized NPs/NEs (2 mg) were dispersed in 2.5 mL phosphate buffered saline (PBS) and ultrasonicated till nanocomposites are dispersed followed by addition of 2 mL MTX and 0.5 mL EDC. The reaction mixture was ultrasonicated for 30 min and centrifuged at 9000 rpm for 10 min followed by three ethanol washes. The resultant Co@Au-SH-MTX (thiol-conjugated Methotrexate) NPs/NEs were then dried under vacuum and characterized using Fourier-transform infrared spectroscopy (FTIR, FT-720, HORIBA, Kyoto, Japan) and Zetasizer (Nano-ZS90, Malvern Instruments Ltd., Holtsville, UK).

### 2.5. Cell Culture

L929 cells (mouse fibroblasts, ATCC-CCL1, ATCC, Manassas, VA, USA), SW620 (colorectal cancer, ATCC-CCL227, ATCC, Manassas, VA, USA) and C6 (glioma, ATCC-CCL107, ATCC, Manassas, VA, USA) were cultured in Dulbecco’s Modified Eagle Media (DMEM) supplemented with 10% FBS, 100 U/mL penicillin and 100 μg/mL streptomycin and cultured in T75 flask. The cells were incubated at 37 °C and 5% CO_2_, 95% humidity atmosphere and passaged every 2 days.

### 2.6. Nanoparticulate Ingestion Analysis Using Bio-Transmission Electron Microscopy (Bio-TEM)

The temporal ingestion of spherical and elliptical nanoparticles by colorectal (SW620) and glioma (C6) cancer cells was analyzed using Bio-TEM. Cells were seeded at a concentration of 5 × 10^4^ cells/mL and allowed to proliferate for 24 h. After the pre-determined time period, NPs and NEs were added into the culture medium at a concentration of 100 μg/mL and harvested after 8 and 12 h. For harvesting, cells were fixed with a mixture of 2% paraformaldehyde and 2.5% glutaraldehyde in 0.1 M cacodylate. After 30 min, the fixatives were aspirated and cells were washed thrice for 15 min each with PBS. The samples were dehydrated and observed using a TEM.

### 2.7. Analysis of Alternating Magnetic Field (AMF)-Induced Hyperthermia

To analyze the ability of NPs and NEs to generate hyperthermia in the presence of an AMF, Co@Au NPs and Co@Au NEs were dispersed in DI water and subjected to high frequency induction waves (HFIW, powercube (HF2) CEIA1.1-2.2 kHz). The starting temperature was maintained at room temperature (26 °C). Co@Au NPs were dispersed in DI water at concentration of 1, 3, 5 and 7 mg/mL whereas the Co@Au NEs were dispersed at a concentration of 5, 10, 15, 20 and 25 mg/mL. The temperature of solution containing nanoparticulates was measured for 10 min at a regular interval of 30 s using an optical fiber thermometer. We used different concentration of NPs and NEs because NEs did not show any change in response to AMF until 20 mg/mL concentration. 

### 2.8. Analysis of Photostimulation-Induced Hyperthermia

To study the efficacy of Co@Au NEs to generate hyperthermia upon photostimulation, 808 nm Laser was used (PSU-III-FDA, Changchun New Industries (CNI), Taipei, Taiwan). The Co@Au NEs were dispersed in DI water at a concentration of 62.5, 125, 250 and 500 μg/mL. The starting temperature was maintained at room temperature (26 °C). The distance between Laser and the samples was fixed at 10 cm. The temperature was measured for 10 min at a regular interval of 30 s.

### 2.9. Evaluation of NPs and NEs Application as Contrast Agents

The applications of Co@Au NPs and Co@Au NEs as Magnetic Resonance Imaging (MRI) and Optical Coherence Tomography (OCT) contrast agents, respectively were evaluated using the following protocol: For MRI application, Co@Au NPs and Co@Au NEs were dissolved in 0.5% Agar at a concentration of 0.4, 0.8, 0.16 and 0.32 mg/mL in a microcentrifuge tube. *T*2 relaxation time was calculated using the equation $S=S_{0e}-\frac{TE}{T2}$ where *S* is the fitted signal, *S*_0_ is the initial amplitude, TE is the echo time. The repetition time (TR) was fixed at 5000 ms and Echo time (TE) was varied from 10 to 320 ms and *T*2 relaxation time was calculated. Transverse relaxation was calculated by reversing the values of *T*2 relaxation time (1/*T*2). The solution was subjected to 3 T nuclear magnetic resonance. The repetition time, TR was 5000 ms and Echo time, TE for Co@Au NPs was 10 to 320 ms and for Co@Au NEs was 60 ms. For OCT application, Co@Au NEs were dissolved in 0.5% acacia at a concentration of 100, 200, 300, 400 and 500 μg/mL and placed in a 24-well plate. The samples were then irradiated with laser light of 1050 nm wavelength followed by OCT imaging. 

### 2.10. In-Vitro Cytotoxicity Analysis of NPs and NEs

The cytotoxicity of Co@Au NPs, Co@Au NEs and Co@Au NEs-MTX was evaluated using L929 fibroblast cell line as a model. L929 cells were seeded at a density of 5 × 10^4^ cells for 24 h. Co@Au NPs, Co@Au NEs and Co@Au NEs-MTX were first sterilized under UV and then added to the cells at a concentration of 100, 200, 300, 400 and 500 μg/mL. The cells were allowed to grow for 24 h after which the cytotoxicity was evaluated using 3-(4,5-dimethylthiazol-2-yl)-2,5-diphenyl tetrazolium bromide tetrazolium reduction assay (MTT). 

To evaluate the efficacy of Co@Au NEs in limiting cancer cell growth, SW620 and C6 glioma cells were first seeded at a concentration of 5 × 10^4^ cells in a 24-well plate for 24 h. Three experimental groups, namely Co@Au NEs, Co@Au NEs-MTX and MTX, were prepared and added to the cells at a concentration of 100, 200, 300, 400 and 500 μg/mL. The cells were allowed to proliferate for 24 h after which the cytotoxicity was evaluated using MTT assay. 

To evaluate the hyperthermia-induced cytotoxicity, SW620 and C6 cells were first seeded in a 24-well plate at a concentration of 5 × 10^4^ cells and allowed to attach for 4 h. Two experimental groups, namely Co@Au NEs and Co@Au NEs-MTX were prepared in a fixed concentration of 500 μg/mL and added to the cells followed by irradiation with laser light of 808 nm wavelength for 1, 3, 5, 7 and 9 min. After hyperthermia treatment, the cytotoxicity was evaluated using MTT assay by measuring absorbance at 562 nm using ELISA reader (F039300, Sunrise Remote, Tecan, Männedorf, Switzerland). For all cytotoxicity experiments, 0.1 g Teflon was used a negative control and 0.1 g latex was used as a positive control and only cell culture media (DMEM) was used as a blank.

### 2.11. Statistical Analysis

All experiments including material characterizations involving size measurement were performed thrice for each experimental group. The cytotoxicity analysis experiments were biologically triplicated. To evaluate the data sets which statistically differed from one another, one-way ANOVA test was used. The level of significance was set as *p* < 0.05 and 0.01. The data were expressed at mean and standard deviations. Significant data sets with *p* < 0.05 were depicted with a * whereas highly significant data sets with *p* < 0.01 and 0.001 were depicted with a ** and ***, respectively. 

## 3. Results and Discussions

### 3.1. Characterization of Co@Au NPs and NEs

**Figure 1:**
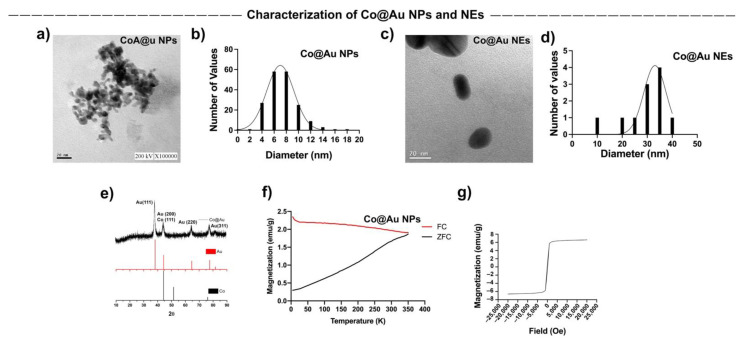
Characterization of round Co@Au nanoparticles (Co@Au NPs) and elliptical Co@Au (Co@Au NEs) nanoparticles. (**a**) TEM micrograph of spherical Co@Au NPs. Scale bar = 20 nm. (**b**) Representation of size distribution of Co@Au NPS. *x*-axis highlights the diameter; *y*-axis highlights the size frequency. The number of particles analyzed was 184. (**c**) TEM micrograph highlighting elliptical-shaped Co@Au NEs. Scale bar = 20 nm. (**d**) Representation of size distribution of Co@Au NEs. *x*-axis highlights the diameter; *y*-axis highlights the size frequency. The number of particles analyzed was 11. (**e**) XRD analysis highlighting 2θ peaks corresponding to crystal structures of Co@Au NPs. (**f**) Analysis of magnetic properties of Co@Au NPs. *x*-axis highlights temperature; *y*-axis highlights magnetization. (**g**) Analysis of magnetic properties of Co@Au NPs. *x*-axis highlights magnetic field strength; *y*-axis highlights magnetization.

Spherical and elliptical-shaped Co@Au nanomaterials were fabricated to study shape-dependent cytotoxicity in mammalian cells. Transmission electron microscope (TEM) was employed to access the nanomaterial morphologies. TEM analysis showed that Co@Au NPs were spherical in shape (Figure 1a) with an average size of 7.46 nm (Figure 1b) whereas the NEs were oval-shaped (Figure 1c) and averagely sized at 29 nm (Figure 1d). Notably, the NEs displayed elliptical geometries which can be attributed to the micro-emulsification route employed in their synthesis which often results in morphological instability. Co@Au NPs were easily oxidized by the atmospheric oxygen resulting in the formation of Cobalt oxide (CoO). Upon oxidation, the color of NPs changes from black to green [29]. In this study, the color of NPs was carefully monitored during the synthesis process. The color of NPs remained black, indicating that oxidation of NPs did not occur. To further confirm this, the nanoparticles were characterized using XRD. The 2θ peaks at 38.1°, 44.33°, 64.64° and 77.5° pertain to (111), (200), (220) and (311) face-centered cubic (FCC) gold planes, indicating the presence of gold. The 2θ peaks at 44.23° relates to (111) FCC plane of cobalt (Figure 1e). The XRD peaks for Co_3_O_4_ are typically present at 31°, 38.2°, 44.4°, 58.3° and 64.6° which pertain to (220), (222), (400), (511) and (440) FCC planes. The absence of any of these peaks in the XRD spectra confirmed that Co@Au NPs were not oxidized during the cleaning process. 

**Figure 2:**
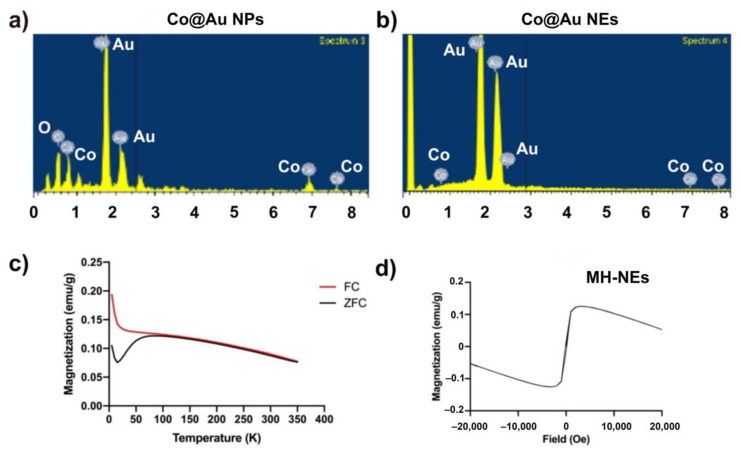
Characterization of round Co@Au nanoparticles (Co@Au NPs) and elliptical Co@Au (Co@Au NEs) nanoparticles composition using EDS and SQUID. (**a**,**b**) Elemental composition analysis of Co@Au NPs and NRs via EDS. (**c**,**d**) Analysis of magnetic properties of Co@Au NRS via SQUID. FC—field-cooled, ZFC—zero-field cooled.

The composition and content ratio of Co@Au NPs NEs were further investigated through Energy Dispersive X-ray spectroscopy (EDS) and inductively coupled plasma Atomic Emission Spectroscopy (ICP-AES) which revealed that the Co@Au NPs were composed of 31.96, 26.07 and 41.98 weight percent oxygen, cobalt and gold, respectively (Figure 2a,b, Table 1). The present of oxygen can be attributed to (i) solvent containing oxygen, (ii) oleic acid which is a stabilizing agent for the NPs, (iii) contaminants inside the instrument and (iv) oxidation of cobalt. However, the XRD analysis confirmed that cobalt was not oxidized. Therefore, the presence of oxygen can be traced back to the other three possibilities. Further characterization revealed that atomic percentages of Co and Au were 16.07 and 8.03, respectively. The composition of NPs was further confirmed using ICP-AES which complemented the EDS results (Table 1). In contrast, the EDS analysis revealed that atomic percentage of Co and Au in Co@Au NEs was 0 and 100%, respectively (Table 1). EDS can only analyze the elements on the surface of the substrate and since Co forms the core of NEs, it is therefore not detected by EDS. Further analysis using ICP-AES displayed that Co@Au NEs were composed of 0.211 and 84.54 wt.% Co and Au, respectively (Table 1). Thus, the elemental ratio was 1:120.

### 3.2. Comparative Analysis of Co@Au NPs and NEs Magnetic Properties

The magnetic properties of Co@Au NPs and NEs were analyzed using superconducting quantum interreference magnetometer (SQUID). The magnetization was measured over temperature ranging from 5 to 350 K. The external magnetic field was fixed at 100 Oe. The ZFC (zero-field cooled) magnetization increases consistently with the increase in temperature. The point of convergence of ZFC/FC (zero-field cooled)/Field-cooled) curves, i.e., the blocking temperature (*T*b) was 350 K which indicated the point of thermal activation (Figure 1f). These results highlight that Co@Au NPs were superparamagnetic in nature at room temperature. The lack of hysteresis can be further attributed to the superparamagnetic property of Co@Au NPs. We then measured the saturation magnetization (Ms) between −20,000 and 20,000 Oe at a room temperature of 300 K which revealed that Ms was 6 emu/g for Co@Au NPs (Figure 1g). In contrast, the ZFC magnetization of Co@Au NEs decreased with temperature elevation (Figure 2c). Furthermore, the magnetization was maintained at 0.1 emu/g (Figure 2d) which further confirmed the Co@Au NPs were more suitable for magnetic field hyperthermia-based applications.

### 3.3. Alternating Magnetic Field (AMF) Induced Hyperthermia by Co@Au NPs and NEs

**Figure 3:**
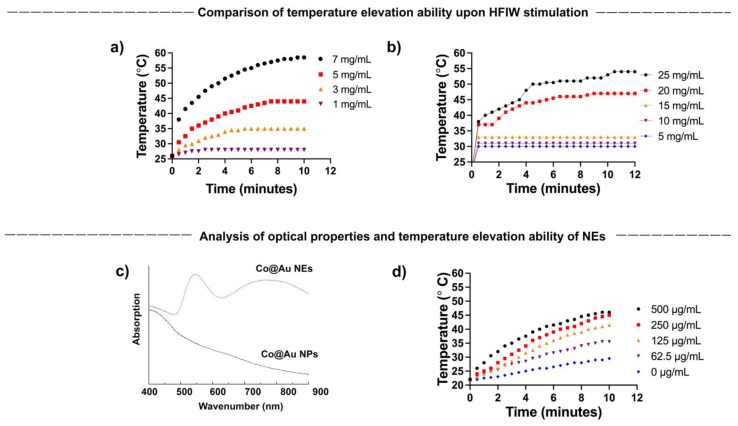
(**a**,**b**) Concentration-dependent hyperthermia ability of round Co@Au nanoparticles (Co@Au NPs) and elliptical Co@Au (Co@Au NEs) nanoparticles. *x*-axis highlights time; *y*-axis temperature (**c**) Analysis of optical properties of Co@Au NPs and NEs via Uv-vis spectrophotometry. (**d**) Concentration-dependent hyperthermia ability of Co@Au NEs upon photostimulation. *x*-axis highlights time; *y*-axis temperature.

The current study aims to establish the shape dependent cytotoxicity of Co@Au NPs and NEs, and in doing so, extend the application of nanomaterials to hyperthermia-induced cancer therapy. To achieve this, we first tested the ability of NPs and NEs to generate hyperthermia upon exposure to AMF. The magnetic property analysis confirmed that the spherical nanoparticles are superparamagnetic in nature. Studies in the past have strongly linked the superparamagnetic nanomaterials to hyperthermia generation. To confirm the behavior in the NPs and NEs synthesized in this study, NPs and NEs were prepared in a variety of concentrations and exposed to AMF. The temperatures were monitored over a period of 10 min. Our results show that the temperature elevation was directly proportional to the NPs and NEs concentration. For NPs, the temperature rose from 26 °C to 42 °C and to 58.5 °C for NPs at 5 and 7 mg/mL concentration, respectively, confirming the ability to trigger hyperthermia upon AFM stimulation (Figure 3a). In contrast, the NEs failed to display a major temperature elevation till the concentration reached 15 mg/mL. The temperature reached 45 °C for 20 mg/mL which was four-fold higher than the NPs concentration (Figure 3b). The results highlight the superior hyperthermia induction by the NPs as compared to the NEs. The reason can be attributed to the higher gold composition in the NEs which results in the decreased magnetocaloric properties.

As shown by the vast number of studies, cellular cytotoxicity is highly dependent on the nanomaterial concentration [30,31]. Collectively, the results suggest that NPs may be a suitable candidate for hyperthermia-induced cancer cell death since a comparatively lower concentration can be employed to reach the target temperature.

### 3.4. Photostimulation-Induced Hyperthermia by Co@Au NEs

A vast number of studies in the past linked the optical properties of nanomaterials to their morphology. We have previously shown that NEs possess better optical properties than NPs owing to their oval morphology [8]. We first tested the optical properties of Co@Au NEs synthesized in this study using UV-Vis. spectrophotometer. Co@Au NPs and NEs were dispersed in absolute alcohol and the absorption peaks were analyzed. Expectedly, Co@Au NPs did not display any absorption peak, confirming the lack of any optical activity (Figure 3c). In contrast, Co@Au NEs displayed a maximum absorption peak at 750 nm, confirming their optical activity (Figure 3c). Furthermore, the optical-excitation of Co@Au NEs also presents an opportunity for photo-stimulation using a laser in near-infrared region (NIR).

We have previously shown that 808 nm laser can be employed to trigger photo-stimulation induced hyperthermia [8]. To test the prospect of using Co@Au NEs for photostimulation induced hyperthermia, Co@Au NEs were prepared in a variety of concentrations ranging from 62.5 to 500 μg/mL and temperature elevation was measured. The results highlighted that upon stimulation with an 808 nm laser, the temperature of medium increased as a function of the NEs concentration. The temperature increased from 22 °C to 41.5 °C at a concentration of 250 μg/mL (Figure 3d). Notably, this concentration was 40-fold lower than the Co@Au NPs concentration which triggered a similar temperature elevation. This result, in particular, makes Co@Au NEs a prospective candidate for hyperthermia utilization to achieve cancer cell death since previously studies have strongly linked cancer cell death at temperatures above 40 °C. Furthermore, the drastic decrease in concentration (500 μg/mL via photostimulation as compared to 25 mg/mL via AMF) to achieve elevated temperature is also expected to trigger less cytotoxicity to non-cancerous cells. However, photostimulation required direct exposure and thus, more therapeutic parameters need to be studied to confirm this. 

### 3.5. Application of Co@Au NPs and NEs as Contrast Agents

**Figure 4:**
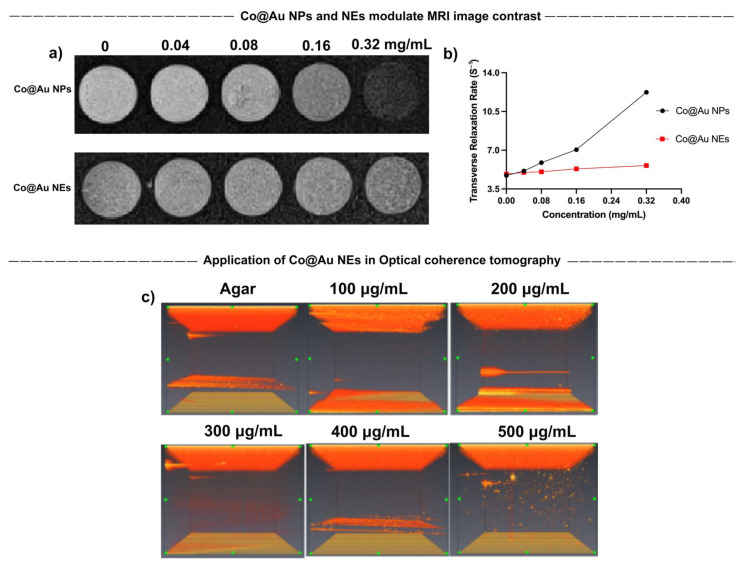
Characterization of round Co@Au nanoparticles (Co@Au NPs) and elliptical Co@Au (Co@Au NEs) nanoparticles as contrast agents in medical imaging. (**a**) MRI images of Co@Au NPs and NEs showing variation in image contrast in a concentration-dependent manner. Co@Au NPs displayed a higher contrast ability than Co@Au NEs. (**b**) Linear regression graph displaying relationship between Co@Au NPs and NEs concentration and transverse relaxation time. (**c**) Optical coherence tomography (OCT) images Co@Au NEs in a concentration-dependent manner. The images show dose-dependent change in contrast, highlighting Co@Au NEs as prospective medical imaging agent.

Magnetic Resonance Imaging (MRI) was performed to evaluate the application of Co@Au NPs and NEs as contrast agents. The results showed that Co@Au NPs were able to induce image darkening and improve contrast with an increase in the concentration which can be attributed to the efficient spin dephasing and *T*2 relaxation (Figure 4a). A linear regression graph was plotted with relaxation rate *r*2 = 23.72 mM^−1^s^−1^ and *R*^2^ = 0.9623. *T*2 relaxation time was maintained at 195.122 ms at a concentration of 0.04 mg/mL which decreased (2.5-fold) to 81.633 at 0.32 mg/mL (Figure 4b). Therefore, a decrease in the *T*2 relaxation time can be attributed for the image contrast enhancement. The results suggest that Co@Au NPs may be employed as suitable contrast agents in medical imaging. Furthermore, Co@Au NEs also displayed a minor modulation in the image contrast. However, this change was less obvious as compared to the Co@Au NPs. The decreased image contrast can be attributed to the higher gold content in the Co@Au NEs, resulting in diminished magnetic properties.

The results of our previous experiments elucidated that Co@Au NEs possess optical properties owing to their morphology. This sparked our interest to exploit its possible application in Optical Coherence Tomography (OCT). Co@Au NEs were prepared in a variety of concentrations ranging from 100 to 500 μg/mL and dispersed in 0.5% agarose gel. Notably, the concentration of Co@Au NEs used here and during hyperthermia analysis were identical. The medium containing Co@Au NEs was irradiated with 1050 nm laser which showed no visible contrast in medium without the NEs. However, a gradual increase in the image contrast was observed as the concentration of NEs increased. Maximum image contrast was observed at 500 μg/mL concentration, highlighting the possibility of using NEs for medical imaging (Figure 4c). Our experiments show that both NPs and NEs can be exploited in medical imaging via different routes. The difference in the methodology of image contrast enhancement stems from the morphological and concentration differences. The results also highlight how nanomaterials can be utilized for same application but via different routes by simply modulating their morphology.

### 3.6. Analysis of Co@Au NPs and NEs Biocompatibility

**Figure 5:**
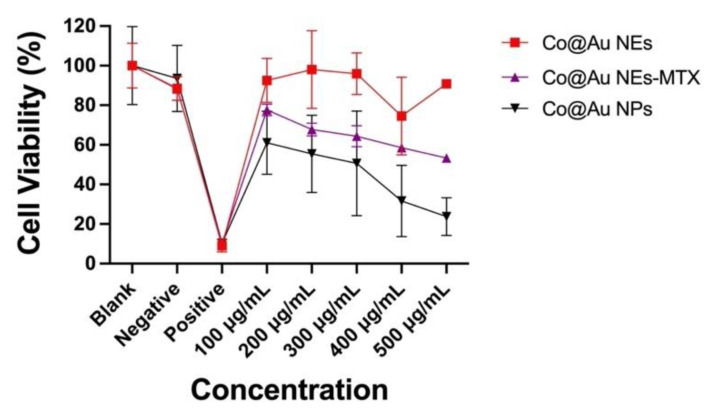
Cytotoxicity analysis of round Co@Au nanoparticles (Co@Au NPs) and elliptical Co@Au (Co@Au NEs) nanoparticles. Cell viability of L929 fibroblasts cultured in varying concentrations of Co@Au NPs, Co@Au NEs and Methotrexate (MTX)-conjugated Co@Au NEs-MTX., 0.1 g Teflon was used a negative control and 0.1 g latex was used as a positive control and only cell culture media (DMEM) was used as a blank.

The primary aim of this study is to elucidate the shape-dependent cytotoxicity of nanomaterials and extend the applications in the field of cancer therapy. To understand this, three experimental groups were prepared, (i) Co@Au NPs (ii) Co@Au NEs and (iii) Co@Au NEs-MTX. MTT assay revealed that viability of L929 fibroblasts was maintained above 90% for NEs at 100 μg/mL concentration which dropped to nearly 85% at 500 μg/mL (Figure 5). In contrast, NPs displayed high cytotoxicity with viability maintained close to 61% at 100 μg/mL concentration which dropped to nearly 20% at 500 μg/mL (Figure 5). Thus, at identical concentrations, NPs were 4-folds more cytotoxic than the NEs which can be attributed to the variation in the nanomaterial shape. This could also be due to the difference in size between NPs and NEs; however, more experiments are needed to validate this. The experimental group containing anti-cancer drug-conjugated Co@Au NEs (Co@Au NEs -MTX) displayed a linear decrease in effect on cell viability due to an increase in the concentration (Figure 5). Notably, this decrease was still not as stark as the one show by NPs, which implies that Co@Au NPs were even more cytotoxic than the anti-cancer drugs. The results of these experiments highlighted that Co@Au NEs were better suited for biological applications than NPs owing to their superior biocompatibility. Therefore, NEs were employed for further experiments. Previous studies have also highlighted the possible role of CTAB in inducing cytotoxicity [32,33]. Thus, the possible role of CTAB in inducing cytotoxicity within the Co@Au NPs experimental group cannot be ruled out, and further experiments must be performed to clarify this. However, given the similarity in the procedure to synthesize NPs and NEs, it is hypothesized that geometry, and not surfactant, is the main reason for higher cytotoxicity of the nanoparticles. 

### 3.7. Confirmation of Co@Au NEs Conjugation with Methotrexate (MTX)

**Figure 6:**
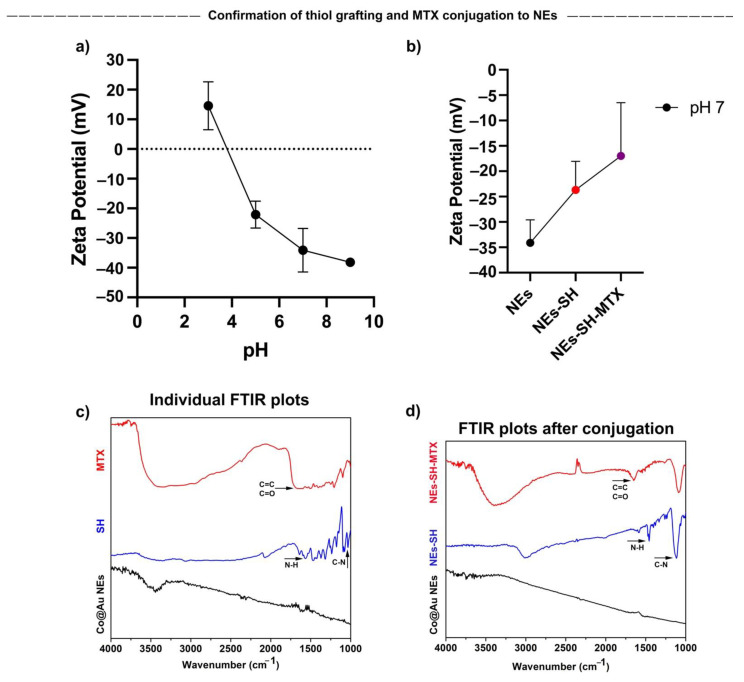
Characterization of elliptical Co@Au (Co@Au NEs) nanoparticles after thiol and MTX (SH-MTX) conjugation via Zetasizer and FTIR. (**a**) Dispersion ability of Co@Au NEs as analyzed by measuring zeta potential at varying pH. (**b**) Measurement of zeta potential after conjugation of Co@Au NEs with thiol (SH) and Methotrexate (MTX). (**c**) FTIR analysis of Co@Au NEs, thiol and MTX. (**d**) FTIR analysis of Co@Au NEs followed by thiol and MTX conjugation, confirming the successful grafting of MTX onto elliptical nanoparticles.

To exploit the applications of Co@Au NEs in cancer therapy, we decided to conjugate Methotrexate, an anti-cancer drug with the NEs. To trigger a homogeneous hyperthermia effect, it is essential that the nanoparticles display high dispersibility. Therefore, before verifying the successful attachment of MTX, we first decided to study the dispersibility of NEs by measuring the surface potential of Co@Au NEs at various pH values (3–9). A decrease in surface potential was observed as the pH increased (Figure 6a). The surface potential was negative at pH 5, 7 and 9. The surface potential at pH 7 and 9 was maintained at −34.1 ± 7.35 and −38.17 ± 0.4, respectively. As described by Bhattacharjee, surface potentials >±30 mV relate to highly stable and easily dispersible nanoparticles, therefore, we can speculate that the NEs are highly stable if used in-vivo [34]. However, further experiments to evaluate their dispersibility in tissues must be performed. Tumor microenvironments display pH range between 6.7 and 7.1 [35], therefore, assessing nanoparticle stability at this pH range in crucial. Our results show that the zeta potential was −34.1 ± 7.35 at pH 7, which highlights the prospect of using Co@Au NEs for in-vivo experiments. 

We then analyzed successful MTX conjugation via Zeta potential measurement. MTX was conjugated to the NEs via 2-aminoethanethiol. The presence of NH_2_ groups are expected to increase the surface potential. Thus, as expected, the zeta potential post-grafting elevated from −34.1 ± 4.51 to −23.7 ± 5.64 mV (Figure 6b). The further elevation in the zeta potential to −17 ± 10.5 after MTX addition confirmed the successful conjugation of MTX, resulting in the formation of Co@Au NEs-MTX complex. 

The successful conjugation of MTX to Co@Au NEs was also confirmed via Fourier transform infrared spectroscopy (FTIR). We first performed FTIR of lone NEs, 2-aminoethanethiol and MTX. The spectra showed the lack of any distinct peak for Co@Au NEs. However, FTIR of 2-aminoethanethiol revealed two distinct peaks at 1640–1450 cm^−1^ and 1350–1000 cm^−1^ which can be attributed to NH and CN bonds, respectively (Figure 6c). Furthermore, FTIR of MTX displayed a stretch at 1680–1600 and 1680–1630 cm^−1^ which can be attributed to C=C and CO bonds, respectively (Figure 6c). We then performed FTIR of Co@Au NEs post-2-aminoethanethiol grafting which displayed the functional group peaks pertaining to N–H and C–N but with higher intensities at 1450 and 1100 cm^−1^, confirming the successful 2-aminoethanethiol grafting (Figure 6d). Upon further MTX conjugation, the C=C and C=O dip emerged which confirmed the successful conjugation of MTX with Co@Au NEs-SH (Figure 6d).

### 3.8. In-Vitro Analysis of Co@Au NEs Ingestion

**Figure 7:**
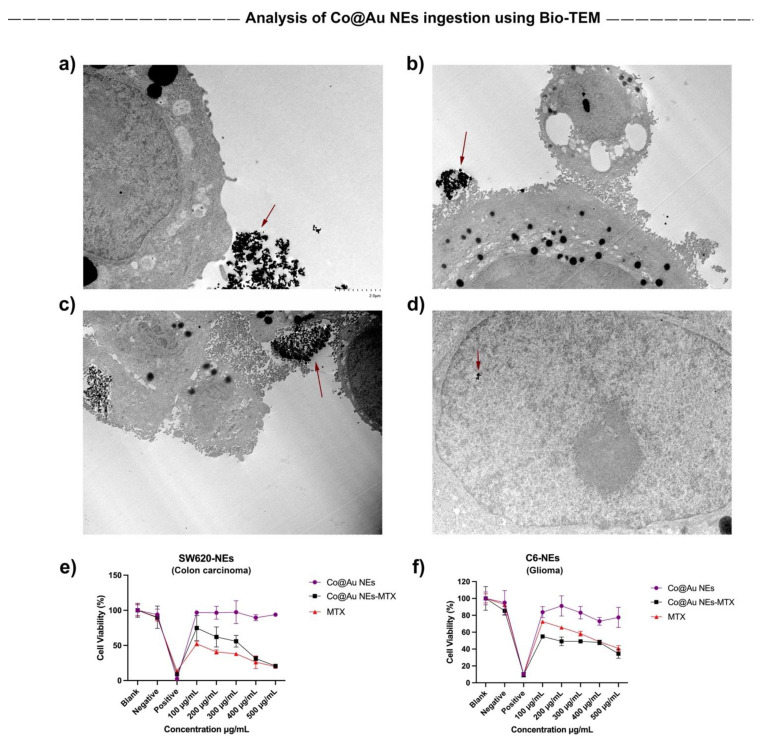
Analysis of elliptical Co@Au (Co@Au NEs) nanoparticle ingestion by SW620 colon carcinoma cells. (**a**–**d**) Bio-TEM micrographs of Co@Au NEs aggregates in SW620 cells after 12-h incubation. The scale bar in figure (**a**–**d**) is 2 µm. (**e**,**f**) Cytotoxicity of Co@Au NEs towards SW620 and C6 glioma cells, respectively. Elliptical nanoparticles displayed higher toxicity towards colon carcinoma cells. Nanoparticle clusters are represented by arrows.

It has been well-studied that the cancer cell surface is negatively charged due to secretion of lactate acid secretion [36,37]. Thus, the nanoparticle endocytosis may depend on nanomaterial size, surface charge, hydrophilicity, and concentration. Furthermore, the nanomaterial ingestion by different cell types is different. To optimize the ingestion time of Co@Au NEs by cancer cells for establishing hyperthermia conditions, we performed Bio-TEM. Notably, NEs possess a negative charge at the surface which may require longer incubations for cellular ingestion. Our results show that after 12 h, Co@Au NEs were present at the cellular surface as well as within the cells, confirming that 12-h incubation of Co@Au NEs is a suitable time period for analysis of photostimulation-based following experiments (Figure 7a–d).

### 3.9. In-Vitro Analysis of Co@Au NEs Interactions with Colon Cancer and Glioma Cells

The results of our previous experiments confirmed that Co@Au NEs display high biocompatibility towards fibroblasts and can be internalized by cancer cells post 12-h incubation. Thus, in the next step, we decided to test the comparative cytotoxicity of Co@Au NEs and MTX-conjugated elliptical nanoparticles (Co@Au NEs-MTX). Our results show that for NEs, the viability of SW620 and C6 glioma cells was maintained above 90% and 80%, respectively at 100 μg/mL concentration (Figure 7e,f). Minimal fluctuation in the viability was observed even when the concentration of the NEs was increased to 500 μg/mL. A consistent decrease in the SW620 cell viability was observed with the increase in the NEs-MTX concentration which can be attributed to the MTX surrounding the NEs (Figure 7e). In contrast, no major difference in the C6 glioma cell viability (60–40%) was observed when the NEs-MTX concentration varied from 100 to 500 μg/mL, implying that the response to drug–nanoparticle conjugate is different to cells of different origin (Figure 7f). Notably, contrary to the expectations, for C6 glioma cells, the viability of experimental group comprising of MTX alone was higher than NEs-MTX. Thus, the drug cytotoxicity is also a function of cell line used. In addition, nanomaterials conjugated to MTX (antifolate type) may be more effective against colon cancers than towards gliomas. Collectively, these results imply that Co@Au NEs engineered in this study are more effective in targeting colon cancer cells than glioma cells and that a single type of nanomaterial may not be equally effective against a wide variety of cancer types.

### 3.10. Photostimulation-Induced Hyperthermia for Limiting Cancer Cell Growth

**Figure 8:**
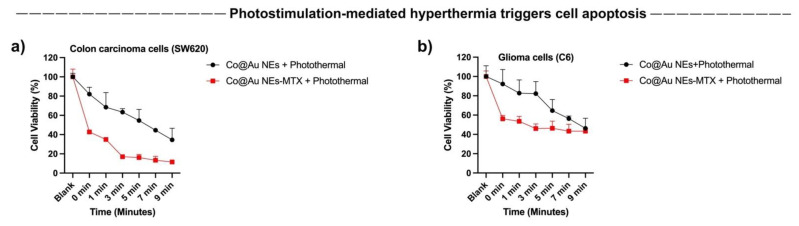
Cytotoxicity of MTX-conjugated elliptical Co@Au (Co@Au NEs) nanoparticles towards SW620 colon carcinoma and C6 glioma cells, respectively, after photostimulation. (**a**,**b**) Photothermal treatment-induced detachment of MTX caused higher cell death in both cell lines (red line); however, the effect was more pronounced in Colon carcinoma cells.

The results of our previous experiments highlighted that Co@Au NEs can generate hyperthermia upon photostimulation. To investigate the effect of photostimulation on cancer cell death and to elucidate if the selective targeting of colon carcinoma cells was consistent, we studied the cancer cell viability by varying the duration of photostimulation and in extension, temperature. Our previous experiments showed that while fibroblasts maintained high viability; however, colon carcinoma cells showed an elevated cell death at 500 μg/mL concentration. Therefore, this concentration was used for photostimulation experiments to access cancer cell death by the elliptical nanoparticles. Our results show that an increment of photostimulation duration triggered a consistent decrease in colon and glioma cancer cells (Figure 8a,b). The cell viability of SW620 cancer cells for the Co@Au NEs experimental group decreased to 80% after 30 s of photothermal stimulation which further declined to 50 and 40% after 7 and 9 min of stimulation, respectively (Figure 8a). In contrast, the SW620 cancer cells displayed an even lower cell viability in NEs-MTX experimental group. The viability was maintained at 40% after 30 s of photothermal stimulation which decreased to 10% after 9 min of stimulation (Figure 8a). The reason behind the superior efficiency of NEs-MTX experimental group in limiting cancer cell growth may be attributed to the detachment of MTX from the nanoparticle–drug conjugate, thereby resulting in a greater cell death.

We repeated similar experiments on C6 Glioma cells and observed a similar trend of cell viability in both experimental groups, i.e., the cell viability declined as a function of exposure to the photothermal stimulation (Figure 8b). For the NEs-MTX experimental group, the C6 glioma cell viability was maintained at 60% after 30 s of stimulation and further decreased to 40% after 9 min of photothermal stimulation (Figure 8b). The percentage dead cells were higher than the lone Co@Au NEs experimental group. Overall, the extent of cell death was lower than what was observed in SW620 cells. The results highlight that the Co@Au NEs can be expected to display a better efficacy in targeting colon carcinoma cells. The reason regarding a greater cell death shown by a specific cancer cell type towards the nanoparticulates is currently now known but will continue to be a focus of the future studies.

## 4. Conclusions

We have engineered Co@Au spherical and elliptical-shaped nanoparticles to highlight the role of nanomaterial geometry on cellular behavior. Our results showed that both Co@Au NPs and NEs can serve as contrast agents and can generate hyperthermia upon application of alternating magnetic field or photostimulation, respectively. However, owing to optical activity, the NEs are more efficient at generating hyperthermia. In-vitro results showed that cells displayed a varying response to both geometries, with a higher biocompatibility towards NEs as compared to NPs. Further experiments showed that NEs can trigger a greater cell death in colon carcinoma cells as compared to glioma cells. Our results showed that upon photostimulation, MTX detaches from Co@Au NEs-MTX complex, resulting in a drastic reduction in cell viability. Collectively, we conclude that cells respond differently to different nanoparticulate shapes and therefore, a rational design of nanomaterial geometry is required while engineering nanomaterials for efficient anti-cancer activity. Applications in the fields of biomedical engineering, cancer biology and theranostics are expected.

## Figures and Tables

**Table 1:** Analysis of Co@Au NPs and Co@NEs composition.

**Quantitative Analysis of Co@Au NPs Elemental Composition via Energy Dispersive X-ray Spectroscopy**		
**Element**	**Weight %**	**Atomic %**
Oxygen	31.96	75.29
Cobalt	26.07	16.67
Gold	41.98	8.03
Total	100	100
**Quantitative Analysis of Co@Au NEs Elemental Composition via Energy Dispersive X-ray Spectroscopy**		
**Element**	**Weight %**	**Atomic %**
Cobalt	0	0
Gold	100	100
Total	100	100
**Analysis of Elemental Composition of Co@Au NPs via Inductively Coupled Plasma Atomic Emission Spectroscopy**		
**Element**	**Weight**	-
Cobalt	28.09	-
Gold	42.63	-
**Analysis of Elemental Composition of Co@Au NEs via Inductively Coupled Plasma Atomic Emission Spectroscopy**		
**Element**	**Weight**	-
Cobalt	0.21	-
Gold	84.54	-

## Data Availability

Data can be available from the authors upon reasonable request.
